# Cardiovascular risk markers associated with arterial calcification in patients with chronic kidney disease Stages 3 and 4

**DOI:** 10.1093/ckj/sfu017

**Published:** 2014-03-09

**Authors:** Chek Ing Kiu Weber, Guillemette Duchateau-Nguyen, Corinne Solier, Annette Schell-Steven, Ricardo Hermosilla, Everson Nogoceke, Geoffrey Block

**Affiliations:** 1 Cardiovascular and Metabolism Disease Therapeutic Area, F. Hoffmann-La Roche Ltd, Basel, Switzerland; 2 Non-Clinical Safety, F. Hoffmann-La Roche Ltd, Basel, Switzerland; 3 Denver Nephrology, Clinical Research, Denver, CO, USA

**Keywords:** arterial calcification, biomarkers, cardiovascular risk, chronic kidney disease, inflammation

## Abstract

**Background:**

The contribution of pro-inflammatory markers to cardiovascular (CV) risk and vascular calcification in chronic kidney disease (CKD) remains largely to be elucidated. We investigated the association between plasma levels of several biomarkers and calcification volume in three different vascular beds in CKD Stages 3 and 4 patients.

**Methods:**

This is a cross-sectional, exploratory study in patients with an estimated glomerular filtration rate (eGFR) ≥20 and ≤45 mL/min/1.73 m^2^ and serum phosphorus ≥3.5 and <6.0 mg/dL enrolled in a previously published randomized, double blind, placebo-controlled single-centre trial. Ethylenediaminetetraacetic acid (EDTA) plasma samples were collected at baseline before patients received study medication and analysed for the presence of a number of biomarkers. Coronary artery calcium (CAC), thoracic aortic calcification (TAC) and abdominal aortic calcification (AAC) volumes were measured using standard electron-beam computed tomography protocols. Associations were adjusted for age, sex, smoking, body mass index, diabetes mellitus status, low-density lipoprotein cholesterol (LDL-C), systolic blood pressure and eGFR.

**Results:**

Associations with CAC were found for β2-microglobulin (B2M), fibroblast growth factor 23 (FGF23), interleukin-8 (IL-8) and IL-18. AAC was associated with: B2M, FGF23 and IL-2 receptor alpha (IL-2 RA). TAC was associated with: B2M, FGF23, IL-2 RA, IL-18 and tumour necrosis factor receptor type I. For most of the analysed biomarkers, there were non-significant trends of associations with calcification.

**Conclusions:**

This exploratory study found that elevated plasma levels of several inflammatory biomarkers are significantly associated with arterial calcification in CKD Stages 3 and 4 patients. A greater understanding of inflammation and calcification in CKD patients may help the development of CV risk-assessment algorithms for better management of these patients.

## Introduction

Chronic kidney disease (CKD) is well known to increase the risk of cardiovascular (CV) disease (CVD) and death [1]. The association between decreased renal function and unfavourable outcomes is so strong that the guidelines of the Kidney Disease Improving Global Outcomes (KDIGO) foundation recommend that all people with CKD should be considered at high risk of CVD [2]. The increased risk of death, CV events and hospitalization is independent of major risk factors, such as a history of CVD or the presence of documented proteinuria [3–5].

The factors and mechanisms underlying the increased CV risk are only partially understood. Traditional CV risk factors including age, diabetes mellitus, hypertension, smoking and dyslipidaemia are prevalent in CKD patients. However, they do not fully account for the increase of CVD and common CVD risk scores may be less accurate in CKD patients than in non-CKD patients [6].

Several non-traditional CV risk factors such as inflammation and abnormalities in mineral metabolism have been identified in CKD [4, 7]. Inflammatory mechanisms appear to be involved in the aetiology of CKD both in animal models and in humans [8], but the role of inflammation in CKD remains unclear. The value of individual pro-inflammatory markers and the mechanisms by which inflammation is associated with different risk profiles remain largely to be elucidated.

Vascular calcification is a common complication of CKD. Coronary artery calcium (CAC) correlates strongly with atherosclerotic plaque burden and the CAC score is a powerful predictor of incidence of CV events and CV risk in addition to traditional risk factors [9–13]. Recent large studies have demonstrated that CAC is associated with estimated glomerular filtration rate (eGFR) in patients with CKD Stages 3 and 4 [14, 15], and a calcium score >100 AU is associated with an 8× increased hazard ratio for long-term cardiac events in patients with Stages 2–5 CKD not requiring dialysis [16].

The role of inflammatory processes in CAC and the associated risk for CVD in CKD remain poorly understood. In the current study, we investigated the association between plasma levels of a number of biomarkers relevant to CKD patients and calcification volume in three different vascular beds in patients with CKD Stages 3 and 4.

## Materials and methods

### Study design

This was a cross-sectional, exploratory study in patients enrolled in a randomized, double blind, placebo-controlled single-centre trial of phosphate binders in patients with moderate to advanced CKD. The main study (ClinicalTrials.gov NCT00785629) was developed and conducted by Denver Nephrology (Denver, CO, USA). Design and results have been reported previously [17].

In the main study, 148 patients with an eGFR ≥20 and ≤45 mL/min/1.73 m^2^ (Modification of Diet in Renal Disease; MDRD formula) and serum phosphorus ≥3.5 and <6.0 mg/dL were recruited between February 2009 and September 2010 and randomly assigned to study treatments or placebo. Exclusion criteria included use of any medication for the purpose of binding intestinal phosphate, use of any active vitamin D or the calcimimetic cinacalcet, intact parathyroid hormone ≥500 pg/mL, or uncontrolled hyperlipidaemia. The exploratory biomarker substudy included 103 patients from the overall study population.

### Blood samples and biomarker analyses

Ethylenediaminetetraacetic acid (EDTA) plasma samples were collected at baseline before patients received any study medication and immediately frozen at −80°C for storage. Frozen EDTA plasma samples were thawed, aliquoted and shipped on dry ice to the respective laboratories, then stored at <−70°C until analysis. We measured levels of a number of inflammatory biomarkers, biomarkers of vascular calcification and associated biological processes and other biomarkers of relevance in CKD patients. A list of the panel of biomarkers measured in EDTA plasma, as well of the methods and responsible measuring laboratories is provided in in Table 1.

**Table 1.** Biomarkers assessed: assay methods and responsible laboratories.

Biomarker	Assay used (laboratory)
β2-Microglobulin (B2M, µg/mL)	RBM Human Custom MAP Core I, HMPC62, HMPC37 (Myriad RBM, Austin, TX, USA)
Fibroblast growth factor 23 C-terminal (FGF23 C-Term, RU/mL)	ELISA (eBioscience, San Diego, CA, USA)
Granulocyte macrophage-colony stimulating factor (GM-CSF, pg/mL)	RBM Human Custom MAP Core I, HMPC62, HMPC37 (Myriad RBM, Austin, TX, USA)
Interferon gamma (IFN gamma, pg/mL)	RBM Human Custom MAP Core I, HMPC62, HMPC37 (Myriad RBM, Austin, TX, USA)
Interleukin-10 (IL-10, pg/mL)	RBM Human Custom MAP Core I, HMPC62, HMPC37 (Myriad RBM, Austin, TX, USA)
Interleukin-18 (IL18, pg/mL)	RBM Human Custom MAP Core I, HMPC62, HMPC37 (Myriad RBM, Austin, TX, USA)
Interleukin-2 (IL-2, pg/mL)	RBM Human Custom MAP Core I, HMPC62, HMPC37 (Myriad RBM, Austin, TX, USA)
Interleukin-2 receptor alpha (IL2.RA, pg/mL)	RBM Human Custom MAP Core I, HMPC62, HMPC37 (Myriad RBM, Austin, TX, USA)
Interleukin-3 (IL-3, pg/mL)	RBM Human Custom MAP Core I, HMPC62, HMPC37 (Myriad RBM, Austin, TX, USA)
Interleukin-4 (IL-4, pg/mL)	RBM Human Custom MAP Core I, HMPC62, HMPC37 (Myriad RBM, Austin, TX, USA)
Interleukin-5 (IL-5, pg/mL)	RBM Human Custom MAP Core I, HMPC62, HMPC37 (Myriad RBM, Austin, TX, USA)
Interleukin-6 (IL-6, pg/mL)	RBM Human Custom MAP Core I, HMPC62, HMPC37 (Myriad RBM, Austin, TX, USA)
Interleukin-7 ( IL-7, pg/mL)	RBM Human Custom MAP Core I, HMPC62, HMPC37 (Myriad RBM, Austin, TX, USA)
Interleukin-8 ( IL8, pg/mL)	RBM Human Custom MAP Core I, HMPC62, HMPC37 (Myriad RBM, Austin, TX, USA)
Latency-associated peptide transforming growth factor β1 (LAP.TGFB1, ng/mL)	RBM Human Custom MAP Core I, HMPC62, HMPC37 (Myriad RBM, Austin, TX, USA)
Monocyte chemotactic protein 1 (MCP1, pg/mL)	RBM Human Custom MAP Core I, HMPC62, HMPC37 (Myriad RBM, Austin, TX, USA)
Osteoprotegerin (OPG, pM)	RBM Human Custom MAP Core I, HMPC62, HMPC37 (Myriad RBM, Austin, TX, USA)
Cystatin C (ng/mL)	RBM Human Custom MAP Core I, HMPC62, (Myriad RBM, Austin, TX, USA)
Eotaxin-2 (pg/mL)	RBM Human Custom MAP Core I, HMPC62, HMPC37 (Myriad RBM, Austin, TX, USA)
Osteopontin (OPN, ng/mL)	RBM Human Custom MAP Core I, HMPC62, HMPC37 (Myriad RBM, Austin, TX, USA)
Major histocompatibility complex Class I-related chain A (MICA, pg/mL)	RBM Human Custom MAP Core I, HMPC62, HMPC37 (Myriad RBM, Austin, TX, USA)
Macrophage inflammatory protein 1 alpha (MIP1ALPHA, pg/mL)	RBM Human Custom MAP Core I, HMPC62, HMPC37 (Myriad RBM, Austin, TX, USA)
Macrophage inflammatory protein 1 beta (MIP1BETA, pg/mL)	RBM Human Custom MAP Core I, HMPC62, HMPC37 (Myriad RBM, Austin, TX, USA)
Matrix metalloproteinase-2 (MMP2, ng/mL)	RBM Human Custom MAP Core I, HMPC62, HMPC37 (Myriad RBM, Austin, TX, USA)
Neutrophil gelatinase-associated lipocalin (NGAL, ng/mL)	RBM Human Custom MAP Core I, HMPC62, HMPC37 (Myriad RBM, Austin, TX, USA)
Tissue inhibitor of metalloproteinases 1 (TIMP1, ng/mL)	RBM Human Custom MAP Core I, HMPC62, HMPC37 (Myriad RBM, Austin, TX, USA)
Tumour necrosis factor receptor Type I (TNFR1, pg/mL)	RBM Human Custom MAP Core I, HMPC62, HMPC37 (Myriad RBM, Austin, TX, USA)
Tumour necrosis factor alpha (TNFALPHA, pg/mL)	RBM Human Custom MAP Core I, HMPC62, HMPC37 (Myriad RBM, Austin, TX, USA)
Tumour necrosis factor beta (TNFBETA, pg/mL)	RBM Human Custom MAP Core I, HMPC62, HMPC37 (Myriad RBM, Austin, TX, USA)

Shaded rows indicate biomarkers that were not included in the analysis because of high variability or low levels.

### Assessment of calcification

CAC, thoracic aortic calcification (TAC) and abdominal aortic calcification (AAC) volumes were measured by electron-beam computed tomography (GE-Imatron C150 scanner) using a standard protocol as previously described [18]. The thoracic aorta was defined as the segment from the aortic root to the diaphragm, whereas the abdominal aorta was defined as the segment from the diaphragm to the iliac bifurcation. The total calcium volume score was calculated as the sum of all lesion volumes [19]. A single experienced investigator performed all image assessments.

### Statistical analysis

Continuous variables are presented descriptively as means and standard deviations (SD) and categorical variables are presented as percentages. Arterial calcification volumes were expressed as median and lower/upper interquartile values. Descriptive data were also calculated for sub-populations according to CKD stages: ≥30 < 60 mL/min/1.73 m^2^ (CKD Stage 3) and <30 mL/min/1.73 m^2^ (CKD Stage 4). For CKD stage comparisons, *t*-tests (all continuous variables except for calcification scores), Kruskall–Wallis tests (calcification scores) or *χ*^2^ tests (categorical variables) were used.

A linear model was used to test the association between elevated levels of a biomarker (independent variable) and each of the clinical assessments: CAC, AAC and TAC, respectively (dependent variable). Due to the presence of outliers in calcifications assessments, a robust linear model was used. The model included the traditional CV risk factors age, sex, smoking, body mass index (BMI), diabetes mellitus status, LDL-cholesterol (LDL-C), systolic blood pressure (BP) and eGFR. Plasma biomarkers and eGFR were log_2_- transformed to enable a normal distribution of data. All calcium volume scores were square-root transformed as is widely done in the field. Clinical assessments and plasma biomarkers were standardized by subtracting the mean and dividing by SD.

Standardized β coefficients of association and 95% confidence intervals (95% CIs) were calculated for biomarker levels and the respective calcification variables and graphically presented in forest plots. A two-tailed *t*-test was performed to test the null hypothesis (*β* = 0, i.e. no association between plasma biomarkers and the clinical assessment). A P-value of 5% was considered as significant.

All analyses were performed with R version 2.13.1.The R package to compare groups (1.0) [20] was used to compare the different CKD stages and the R package rmeta (version 2.16) for generating forest plots.

## Results

### General characteristics of the study population

The studied population comprised 103 patients with CKD Stage 3 (eGFR ≥30 < 60 mL/min/1.73 m^2^) and CKD Stage 4 (<30 mL/min/1.73 m^2^). Baseline characteristics are summarized in Table 2.

**Table 2.** Demographic characteristics of the patient cohort

	All (*N* = 103)	CKD = 3 (*N* = 56)	CKD = 4 (*N* = 47)	P-value^a^
Sex female, *N* (%)	51 (49.5%)	29 (51.8%)	22 (46.8%)	0.76
Age, years, mean (SD)	67.0 (11.0)	66.1 (12.2)	68.0 (9.33)	0.373
Ethnicity Caucasian, *N* (%)	88 (85.4%)	50 (89.3%)	38 (80.9%)	0.353
BMI, kg/m^2^ mean (SD)	31.5 (7.17)	30.7 (6.27)	32.5 (8.08)	0.213
Hypertension,^b^ *N* (%)	102.0 (99.0%)	56.0 (100%)	46.0 (97.9%)	0.456
Diabetes mellitus^c^ *N* (%)	54.0 (52.4%)	28.0 (50.0%)	26.0 (55.3%)	0.734
Smokers^d^, *N* (%)	58 (56.3%)	31 (55.4%)	27 (57.4%)	0.99
LDL-cholesterol,^e^ mg/dL, mean (SD)	95.5 (32.9)	91.7 (28.7)	100.0 (37.1)	0.215
Systolic BP, mmHg, mean (SD)	127 (16.1)	124 (15.1)	130 (16.9)	0.111
eGFR, mL/min/1.73 m^2^, mean (SD)	31.2 (8.39)	37.2 (6.17)	24.0 (3.53)	<0.001
CAC volume score,^f^ mm^3^, median [1st quartile; 3rd quartile]	234.0 [43.0; 737]	222.0 [79.2; 738]	246.0 [27.0; 661]	0.768
AAC volume score,^f^ mm^3^, median [1st quartile; 3rd quartile]	1623 [258; 5377]	1254 [269; 5114]	2030 [212; 5377]	0.478
TAC volume score,^g^ mm^3^, median [1st quartile; 3rd quartile]	501 [104; 2104]	418 [74.0; 2083]	867 [150; 2040]	0.476
Patients with measurable AAC volume score at baseline, *N* (%)	81 (87.1%)	45 (86.5%)	36 (87.8%)	0.99
Patients with measurable CAC volume score at baseline, *N* (%)	78 (83.9%)	46 (88.5%)	32 (78%)	0.284
Patients with measurable TAC volume score at baseline, *N* (%)	65 (82.3%)	36 (81.8%)	29 (82.9%)	0.99

^a^P-value for the comparison of CKD Stage 3 with CKD Stage 4.

^b^Measured in patients with hypertension only.

^c^Measured in patients with diabetes mellitus only.

^d^Smokers include patients who currently smoke or have ever smoked.

^e^*N* = 102.

^f^*N* = 93.

^g^*N* = 79.

The mean age was 67 years (SD = 11), evenly distributed within both CKD stages. Women represented nearly half of the study population in each CKD stage. The majority of patients were Caucasian (85.4%). Mean BMI was 31.5, similar in both CKD stages. Nearly half of the population had diabetes mellitus, equally distributed in each CKD stage. All except one patient had hypertension (99%). The mean systolic BP was 127 mmHg with no difference between CKD Stages 3 and 4. Half of the patients were smokers (defined as current smokers and have ever smoked).

CAC, TAC and AAC volumes are summarized in Table 2. Approximately 80% of patients had measurable calcification at baseline, and there was no significant difference between the CKD Stages 3 and 4. The median calcium scores were 234, 501 and 1623 mm^3^ for CAC, AAC and TAC, respectively. There were no significant differences in average calcification score values between CKD stages in any of the three vascular beds.

### Levels of screened plasma biomarkers

The levels of 8 of the 29 biomarkers measured were below the limit of quantification in all (or nearly all) samples. Three biomarkers (interferon gamma, tumour necrosis factor alpha, interleukin-10) were excluded from the analysis as they were exhibiting high variability between technical replicates. Table 3 summarizes the levels of plasma biomarkers in patients with CKD Stages 3 and 4, respectively. A number of markers showed a significantly increased plasma levels in patients with greater severity of disease: β2-microglobulin (B2M), fibroblast growth factor 23 (FGF23), interleukin-2 receptor alpha (IL-2 RA), osteoprotegerin, cystatin C, osteopontin, neutrophil gelatinase-associated lipocalin (NGAL) and tumour necrosis factor receptor Type I (TNFR1). For the other analysed biomarkers, no correlation between biomarker levels and severity of CKD was observed.

**Table 3.** Mean (SD) plasma levels of assayed biomarkers in the study cohort by CKD severity

Biomarker	Mean (SD) concentrations		P-value
	CKD = 3 (*N* = 56)	CKD = 4 (*N* = 47)	
β2-Microglobulin (µg/mL)	4.43 (1.06)	6.46 (2.13)	<0.001
Fibroblast growth factor 23 c-terminal (RU/mL)	200 (157)	327 (248)	0.003
Interleukin-18 (pg/mL)	180 (95.7)	185 (77.1)	0.798
Interleukin-2 receptor (pg/mL)	3602 (1179)	4253 (1557)	0.021
Interleukin-8 (pg/mL)	7.61 (3.77)	7.25 (4.67)	0.682
Latency-associated peptide transforming growth factor β1 (ng/mL)	5.33 (3.60)	4.81 (2.91)	0.422
Monocyte chemotactic protein 1 (pg/mL)	97.3 (35.7)	91.8 (24.2)	0.357
Osteoprotegerin (pM)	7.06 (2.67)	8.40 (2.95)	0.018
Cystatin C (ng/mL)	1679 (329)	2200 (490)	<0.001
Eotaxin-2 (pg/mL)	807 (720)	765 (582)	0.749
Osteopontin (ng/mL)	21.9 (11.8)	29.9 (20.4)	0.019
Major histocompatibility complex Class I-related chain A (pg/mL)	138 (75.7)	132 (88.2)	0.778
Macrophage inflammatory protein 1 beta (pg/mL)	155 (83.0)	161 (166)	0.841
Matrix metalloproteinase-2 (ng/mL)	1152 (336)	1143 (277)	0.884
Neutrophil gelatinase-associated lipocalin (ng/mL)	312 (103)	431 (143)	<0.001
Tissue inhibitor of metalloproteinases 1 (ng/mL)	88.8 (22.7)	96.6 (22.7)	0.085
Tumour necrosis factor receptor Type I (pg/mL)	4450 (1876)	6393 (2067)	<0.001

### Association of biomarkers and calcification in different vascular beds

**Fig. 1.**
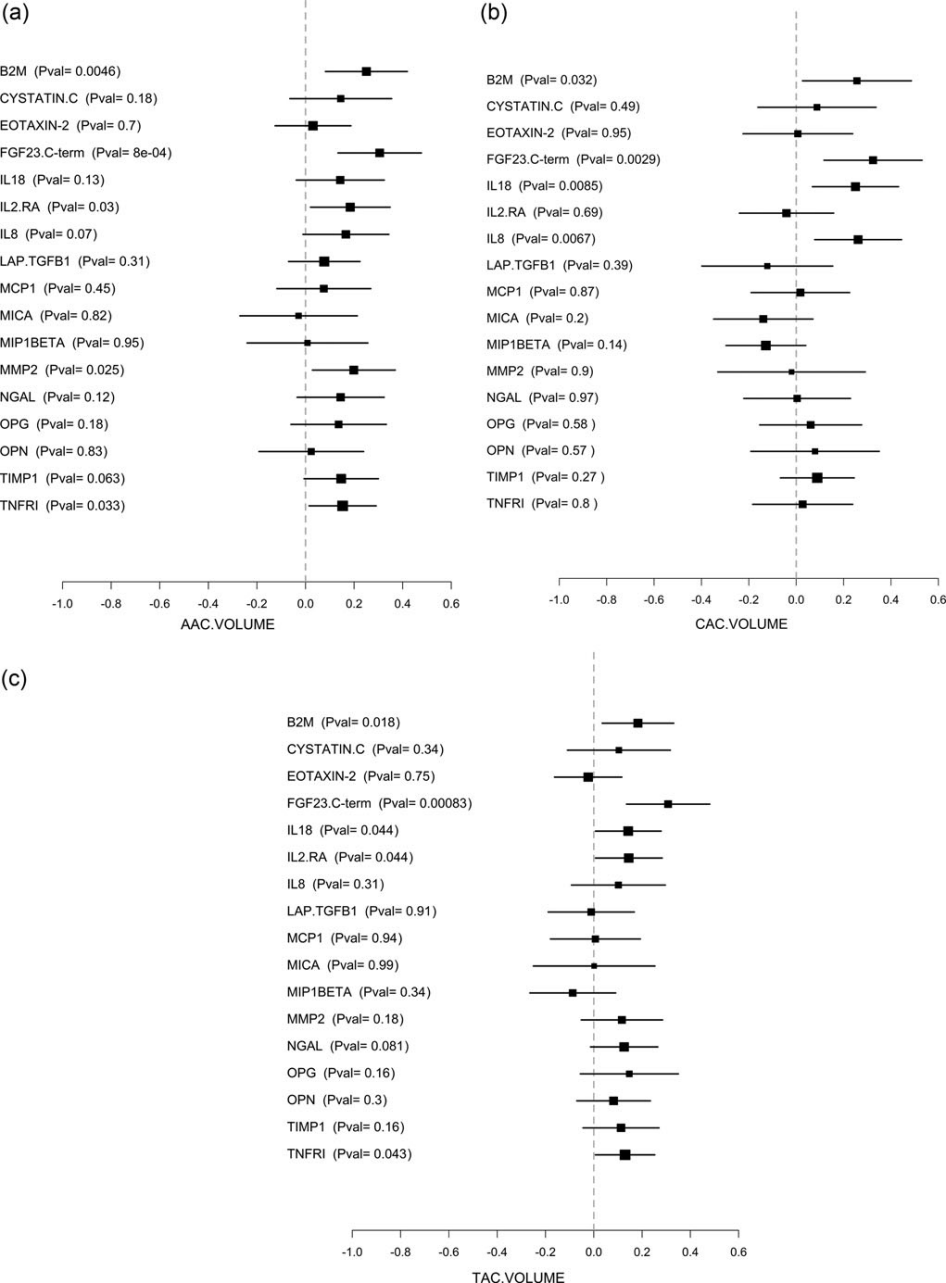
Standardized coefficient of association and 95% CIs between plasma levels of the assayed biomarkers and (**a**) CAC volume; (**b**) AAC volume; (**c**) TAC volume, respectively. The size of the black box indicates sample size. B2M, β2-microglobulin; FGF23, fibroblast growth factor 23 C-terminal; IL-18, interleukin-18; IL-8, interleukin-8; IL2.RA, interleukin-2 receptor alpha; LAP.TGFB1, latency-associated peptide transforming growth factor β1; MCP1, monocyte chemotactic protein 1; OPG, osteoprotegerin; OPN, osteopontin; MICA, major histocompatibility complex Class I-related chain A; MIP1BETA, macrophage inflammatory protein 1 beta; MMP2, matrix metalloproteinase-2; NGAL, neutrophil gelatinase-associated lipocalin; TIMP1, tissue inhibitor of metalloproteinases 1; TNFRI, tumour necrosis factor receptor Type I.

Association coefficients and 95% CIs between the analysed plasma biomarkers and CAC, AAC and TAC after adjustment for traditional risk factors are shown in Figure 1a–c, respectively. Associations with CAC were found for B2M, FGF23, IL-8 and IL-18. AAC was associated with B2M, FGF23 and IL-2 RA. An association with TAC was found for B2M, FGF23, IL-2 RA, IL-18 and TNFRI. Non-significant trends towards association with calcification were found for most of the other analysed biomarkers. Only for major histocompatibility complex, Class I-related chain A (MICA) and macrophage inflammatory protein 1-β were (non-significant) trends towards lesser CAC volume scores observed.

## Discussion

The refinement of strategies for CV risk evaluation in patients with CKD remains a major task in order to better characterize patients at risk and ensure appropriate therapies to reduce clinical outcomes. This study supports the published evidence that arterial calcification is a common finding in CKD Stages 3 and 4 patients, i.e. ∼80% of patients had measurable calcification volume in the three vascular beds. The results presented here also suggest that elevated plasma levels of several inflammatory biomarkers are statistically significantly associated with calcification volumes in three selected arterial beds in CKD Stages 3 and 4 patients, even after adjusting for the traditional risk factors age, sex, smoking, BMI, diabetes mellitus status, LDL-C, systolic BP and eGFR.

The results add to the growing body of evidence supporting the role of inflammation in CVD. A number of circulating pro-inflammatory cytokines are established as risk markers for CVD and increased plasma levels of markers of inflammation predict the risk of developing CKD [8]. However, the value of individual pro-inflammatory cytokines in different patient populations and the mechanisms by which different inflammatory processes are associated with CV risk profiles remain largely to be elucidated. Calcification in different vascular beds has been shown to be associated with higher risk of total, non-CVD and CVD mortality in unselected patient populations [21]. To our knowledge, this is the first study to investigate a large number of biomarkers for the correlation with arterial calcification in a population with CKD.

We analysed the association of the biomarkers with CAC, AAC and TAC. Of these calcium scores, the predictive value of CAC for future occurrence of fatal and non-fatal CV events has been demonstrated in a number of studies in epidemiological cohorts [9, 16, 22]. In CKD Stages 3 and 4 populations, similar to the current study, CAC is associated with eGFR [14, 15], although the reverse is not necessarily the case. Calcification in the thoracic and abdominal aorta may be less valuable as an independent marker of risk. For TAC, a strong association with total mortality has been reported, but the association with CVD mortality was weaker than that reported for CAC [21]. We note that, in the present study, the greatest number of associated inflammatory biomarkers was found for CAC, with fewer associations between biomarkers and AAC or TAC.

B2M and FGF23 were significantly associated with calcification in all three vascular beds. Elevated serum levels of B2M are known to correlate with an inflammatory response during haemodialysis [23]. In addition, B2M is a predictor of peripheral artery disease [24], and increased B2M levels are significantly associated with adverse CV outcome in patients with prevalent asymptomatic carotid atherosclerosis [25]. Recently, Liabeuf *et al*. [26] found that B2M levels >8.34 mg/L were a significant predictor of overall and CV mortality in a cohort of patients at different stages of CKD. Notably, the investigators found a significant, linear association between B2M levels and (abdominal) aortic calcification score.

FGF23 is known to be a sensitive marker of impaired kidney function, with elevations preceding detectable increases in serum creatinine [27]. In subjects with normal kidney function, FGF23 is not associated with CAC [28] and levels are thought to increase as a compensatory response to maintain normal phosphate balance as the capacity for renal phosphorus excretion declines [29]. However, the relationship between FGF23 and calcification in moderate CKD remains controversial. An association of FGF23 with AAC has been reported in older men [30]. In patients on haemodialysis, some studies have reported an independent positive correlation between FGF23 and peripheral and aortic calcification [31, 32]. Similar conflicting reports exist for the association between FGF23 and CAC in pre-dialysis CKD patients: Gutiérrez *et al*. [33] found no association between FGF23 and CAC in pre-dialysis patients in a multivariate analysis, whereas Nakayama *et al*. [34] reported FGF23 to be independently related to CAC in patients even at Stages 1 and 2 [34]. Our findings support the latter results but the predictive value of this association remains to be determined.

Interleukins are extensively studied biomarkers of inflammation in CVD and the plasma levels of many interleukins, IL 6, IL-12, IL-18 among them, have been shown to be elevated in later stages of CKD [7, 35]. IL-6 levels predict the risk of developing incident CKD [8] as well as CVD and mortality in patients with end-stage renal disease [36]. Links between elevated plasma levels of cytokines and calcification in less advanced CKD have yet to be demonstrated. In the current work, increases in plasma levels of both IL-8 and IL-18 were significantly associated with the CAC score, but an association between increased plasma levels and the TAC score was found only for IL-18, and no association was found for the AAC score. The role of IL-8 as a CVD risk marker appears to have been studied less than IL-6 and IL-18 and further investigation is needed.

Several limitations to the study should be noted. It was an exploratory, retrospective analysis from a single-centre study in the USA and the generalizability of our findings to other geographic regions and populations may be limited. The correlations we identified were to surrogate markers of CVD and we did not correct the borders of statistical significance for multiple comparisons. The predictive value of the markers on CV outcomes and/or CKD progression, as well as any causative mechanisms, would need to be demonstrated in appropriately designed studies. Although both calcification and inflammation have been shown to predict CV risk in cohort studies, several studies have failed to show additional predictive value of inflammatory markers (typically C-reactive protein) when added to CAC (reviewed by Hamirani *et al*. [37]). Whether any of the inflammatory markers studied here would be an independent marker of CV risk in a population with CKD and CAC likewise remains to be established.

In summary, we found that elevated plasma levels of several inflammatory biomarkers are significantly associated with arterial calcification in CKD Stages 3 and 4 patients, even after adjusting for traditional risk factors such as age, sex, smoking, BMI, diabetes mellitus status, LDL-C, systolic BP and eGFR. These findings may provide supporting information for two lines of research: the understanding of calcification, specifically in CKD patients, and the development of improved algorithms for risk assessment and tailored therapies in these patients.

## Funding

This work is fully funded by F. Hoffmann-La Roche Ltd.

## Conflict of interest statement

None declared.
